# Effect of social order, perch, and dust-bath allocation on behavior in laying hens

**DOI:** 10.5713/ab.21.0198

**Published:** 2021-06-24

**Authors:** Yanan Wang, Runxiang Zhang, Lisha Wang, Jianhong Li, Yingying Su, Xiang Li, Jun Bao

**Affiliations:** 1 College of Animal Science and Technology, Northeast Agricultural University, 150030 Harbin, China; 2 College of Life Science, Northeast Agricultural University, 150030 Harbin, China; 3 Key Laboratory of Chicken Genetics and Breeding, Ministry of Agricultural and Rural, 150030 Harbin, China

**Keywords:** Behavior, Dust-bath, Laying Hen, Perch, Social Order

## Abstract

**Objective:**

The objective of this study was to determine the effects of different social ranking order (SRO) and the enrichments (perch and dust-bath) allocation (EA) on behavior of laying hens in furnished cages.

**Methods:**

Total experimental period was 4 weeks. There were 216 Hy-line brown layers beak-trimmed at 1 d of age and selected randomly at 14 weeks of age from a commercial farm, and randomly divided into 36 cages with 6 hens in each cage. High enrichments (perch and dust-bath) allocation (HEA) and low enrichments (perch and dust-bath) allocation (LEA) were provided. Video observations of behavior were obtained from the focal hens between 14 and 18 weeks of age and perching, dust-bathing and other general behaviors of the hens with different social orders were measured.

**Results:**

Perching behavior of high SRO hens (HSR) were significantly higher than that of medium SRO hens (MSR), and that of the MSR were significantly higher than that of low SRO hens (LSR) (p<0.01), except for lying on perch (p>0.05). The hens in the high EA cage (HEAC) showed more lying behavior on perch than those in the low EA cage (LEAC) (p< 0.01). The different SRO and EA did not affect dust-bathing behavior except vertical wing-shaking behavior (p<0.05). The LEA did not affect general behaviors (p>0.05), except standing and preening behaviors (p<0.01 and p<0.05), of which the hens in the HEAC showed less standing (p<0.01) and more preening behavior than the hens in the LEAC.

**Conclusion:**

The SRO of laying hens has a significant effect on the perching behaviors, but SRO and EA have little effect on dust-bathing and general behaviors.

## INTRODUCTION

In natural or semi-natural conditions, chickens live in defined social groups, using auditory and visual stimuli to learn social behavior from each other [1]. Laying hens’ social nature may be affected when they are reared in traditional cages with limited space. Furnished cages are designed to encourage hen motivation to perform natural behaviors, such as perching, dustbathing, and nesting for improving their welfare, and provide a condition allowing individual competition among laying hens for enrichments as compared to conventional cages. When the enrichments (perch and dust-bath) allocation (EA) is insufficient, the individual social ranking order (SRO) determines its superiority in enrichments competition among individuals [2]. Banks et al [2] reported that perches were occupied by the inferiors during the daytime, possibly to evade the attack of dominant hens. Shimmura et al [3,4] studied the competition of dust-baths in furnished cages and reported that the dominant hens had the priority in choosing dust-bathes, while the average dust-bathing time of the inferior hens were shorter than the dominant or middle hens [5]. Besides that, the social competition of laying hens was also manifested in perch competition, and the dominant individuals tended to expel the inferior hens during nighttime rest [6]. It could be seen that the dominant hens had the privilege to use enrichments [7,8].

In addition, the social nature of laying hens has its facilitating effect on behavioral expression. Dust-bathing behavior has social promotion effect [9] and show dust-bathing synchronization. When one chicken starts to perform dust bathing, other layers will join in [10]. Duncan et al [11] found that laying hens expressed more dust-bathing behavior when they saw the companions taking dust-bath. However, the research results from Barnett and Hemsworth [12] reported that dust-bathing behavior was not affected by the social promotion of other layers. Some social factors, such as imitation, social facilitation etc., would have an impact on the perching behavior of hens [13].

Social hierarchy is an important factor of individual adaptation in a group, which affects territory possession, mating success rate, reproduction and survival [14]. Chickens are gregarious animals, and their population dominance sequence is established after pecking, the factors that affect the fighting behavior are enrichments, stocking density or the group size [2,15]. The study conducted by Shimmura et al [16] showed that the dominant hens in a relatively large group (18 birds/cage) performed more dust-bathing or nesting behavior than the inferiors, whereas there was no obvious individual difference in a relatively small group (5 birds/cage).

There is evidence for a relationship between enrichments competition and the allocation. If there is a deficiency in the allocation of enrichments, the individual’s occupancy of enrichments will be affected even if there is no competition, such as the lack of nest box would increase eggs laying outside the nest [17]. At present, it is unclear about how the social competition of laying hens affects the utilization of enrichments in the furnished cages as allocation of enrichments is varying. Therefore, this study aimed to investigate the effects of SRO of laying hens on the behavior and the utilization of the enrichments and attempted to understand the relationship between the SRO of laying hens and EA in small furnished cages.

## MATERIALS AND METHODS

### Ethics statement

All experiments were conducted according to the guidelines of the Institutional Animal Care and Use Committee of Northeast Agriculture University (NEAU- [2011] -9).

### Experimental animals and management

A total of 216 Hy-line brown layers beak-trimmed at 1 d of age were selected randomly at 14 weeks of age from a commercial farm, and randomly divided into 36 cages with 6 hens in each cage, and whether the birds in each cage knew each other was not considered. The size of the cages was 100 cm×50 cm×70 cm, providing a stocking density of 833 cm^2^/hen (higher than the EU requirement at least 750 cm^2^/hen). All laying hens were kept in an enclosed test house with mechanical ventilation, allowing room temperature to vary from 18°C to 24°C and humidity from 40% to 70%. Artificial lighting was on for 9 hours starting from 7:00 h to 16:00 h with a light intensity of 5 to 10 lux. All birds had no experience of using perch and dust-bath before the experiment. The hens received a commercial diet (metabolizable energy of 11.87 MJ/kg, crude protein of 15.50%) and were fed *ad libitum* each day, water was available ad libitum in all cages. The experiment started at 14 weeks of age and finished at 18 weeks of age.

### Perch and dust bath allocation

All cages were subjected to 2 levels of EA: high and low EA levels. The high enrichment allocation cage (HEAC) were equipped with two vertical perches consisting of a long perch (100 cm long and 20 cm away from the bottom floor of cage) and a short perch (50 cm long and 40 cm away from the bottom floor of cage), providing 25 cm perch per hen and two dust-bathing areas (25 cm×50 cm×5 cm with 5 cm thick sand), allocated at the bottom of the two sides of the cage. The low enrichment allocation cage (LEAC) were equipped with one perch (90 cm long and 20 cm away from the bottom floor of cage, 15 cm perch per hen) and one dust-bath area (25 cm×50 cm×5 cm, with 5 cm thick sand), located at one side of the cage.

### Behavioral observation

The behaviors were recorded with a surveillance system (4 mm infrared network cameras, DS-2CD3210D-15, hardware recorder, DS-7816N-E2, Hangzhou Hik-vision Digital Technology Co., Ltd., Hangzhou, China) located at the opposite side of the cages which was 0.5m away from the target cages with full view of the cages. All hens from each cage were observed for behavioral collection and leg tags with 6 different colors (white, blue, red, yellow, orange, and green) were used for individual identification. The behavioral observation was started on the fourth day after 3 days acclimation to the new cage environment. Aggressive behavioral data were collected from 14 to 18 weeks of age for two periods of the observation time (08:30 to 10:30 h and 13:00 to 15:00 h) in each observing day (2 days/week), and each group was observed for 40 hours. The aggressive behavior parameters included pecking, displacing, chasing, and threatening and are listed in Table 1 and they were recorded with continuous recording method [18,19]. Clutton-Brock index (CBI) of fighting success was used to determine the SRO of an individual among a cage [20]. All aggressive behaviors of each hen over two observational periods were pooled and recorded as the total number of its win (W) or loss (L).

According to the results of CBI of each individual, the social orders of the individual laying hens in each group were determined according to the methods of Shimmura et al [19]. Briefly, the 1st and 2nd higher CBI index hens were regarded as the high SRO hens (HSR), and the 3rd and 4th higher CBI index hens were as the medium SRO hens (MSR) and the 5th and 6th CBI index hens were as the low SRO hens (LSR). Therefore, the experiment was a design of 2×3 factors (2 levels of resource allocations and 3 levels of SRO).

The study mainly observed the perching, dustbathing and also observed general behaviors (listed in Tables 2 to 4) were sampled from 14 to 18 weeks of age and were recorded continuously for two periods (08:30 to 10:30 h and 13:00 to 15:00 h) in each observing day (2 days/week). For perching, dust-bathing behaviors and general behaviors, continuous recording and one-zero sampling were used for the focal hens on the perches and at the dust-bath. The state behaviors were represented in percentages (converting the behavior data into a percentage of the total observation time.), for the event behaviors, each incident behavior was recorded as one time, which was expressed by frequencies (the total number of occurrences per minute) [21]. The perching behaviors and the dustbathing behaviors were recorded separately and divided into event behaviors and state behaviors. Perching event behaviors were included preening, staring, exploring and comforting behavior, perching state behaviors were included standing, lying and walking behavior. Dustbathing event behaviors were included bill raking, pecking, vertical wing shaking, side scratching, vigorous body shaking and body movement behavior, dustbathing state behaviors were included head forward and head under wing behavior. The general behaviors were also divided into two categories. One was general state behaviors which included feeding, standing, lying, walking, perching and dustbathing, and the other was general event behaviors which included drinking, preening, staring, pecking, comforting, head shaking feather pecking and escaping behavior. All behavioral observation was conducted by the two experienced technicians.

### Statistical analysis

Data were processed with Excel 2003 (Microsoft Corporation, 2003). All behavioral data were examined for normal distribution (Kolmogorov-Smimov procedure). Those data of drinking, pecking, comforting, feather pecking, lying, exploring and pecking and body movement were found not normally distributed and they were transformed with Square Root Transformation. All data (including those transformed data) were analyzed by the Statistic Package for Social Science (SPSS 23.0; software IBM Institute Inc, Chicago, IL, USA).

The analysis of the effect on behavior of hens by SRO and EA was subjected to the analysis of variance using Factorial-ANOVA analysis procedure, the following formula was used as Y_ijk_ = μ+α_i_+β_j_+(αβ)_ij_+γ_k_+e, where Y_ijk_ as total individual observation value, μ as population mean, α_i_ as effect of SRO, β_j_ as effect of EA, (αβ)_ij_ as interaction between SRO and EA, γ_k_ as block effect, e as random error. And the differences between the effects were tested by Duncan’s multiple range tests. SRO and EA were set as two main effects. p≤0.05 presented as the difference is significant, and p≤0.01 presented as the difference is highly significant. The values in the text are expressed as mean±standard deviation (SD).

## RESULTS

### Effects of social ranking order, perch and dust-bath allocation on perching behavior

Mean (SD) dominance index of HSR, MSR, and LSR were 0.917±0.02, 0.841±0.022, and 0.753±0.032 in HEAC, 0.921± 0.034, 0.835±0.022, and 0.742±0.075 in LEAC, the linear correlation coefficient R^2^ were 0.998 and 0.999 respectively.

As it is shown in Table 5, SRO had a significant effect on standing, preening, walking, staring, exploring, and comforting behavior on perch (all p<0.01) except for lying behavior (p = 0.19). Among them, the standing behavior on perch in HSR was significantly higher than that of MSR and LSR (p< 0.01), while there was no significant difference between MSR and LSR in standing behavior. The behaviors of walking, preening, staring, exploring, and comforting on perch showed that the perching behaviors were in the order: HSR> MSR>LSR (p<0.01). Different from the SRO, the hens in HEAC showed more lying behavior on perch than those in LEAC (p<0.01), but there was no effect on any other perching behavior between the two resource allocations (all p> 0.05).

### Effects of social ranking order, perch and dust-bath allocation on dust bathing behavior

**Figure 1:**
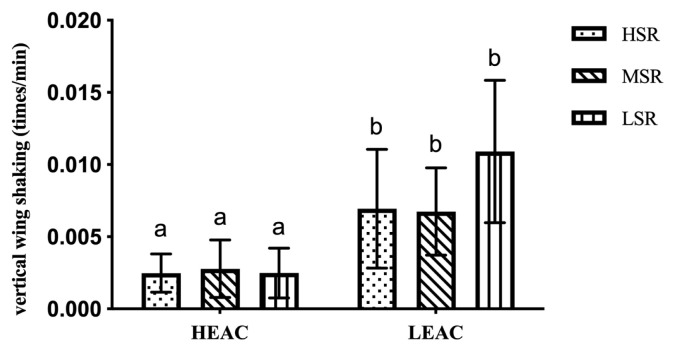
Effects of hen’s social ranking order (SRO) on vertical wing shaking in high and low enrichments allocation cages (HEAC and LEAC). ^a,b^ p<0.01.

The EA did not affect dust bathing behavior of the hens at the dust box (all p>0.05) except vertical wing shaking (p< 0.05), which showed less in HEAC than LEAC. While SRO had no significant effect on all the dust bathing behavior of the hens (as shown in Figure 1).

### Effects of social ranking order, perch and dust-bath allocation on general behavior

**Figure 2:**
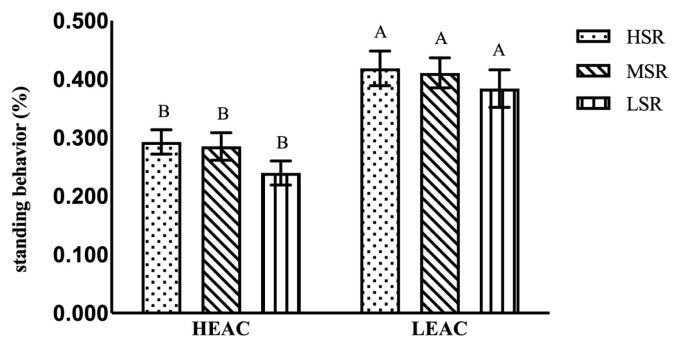
Effects of hen’s social ranking order (SRO) on standing behavior in high and low enrichments allocation cages (HEAC and LEAC). ^A,B^ p<0.001.

**Figure 3:**
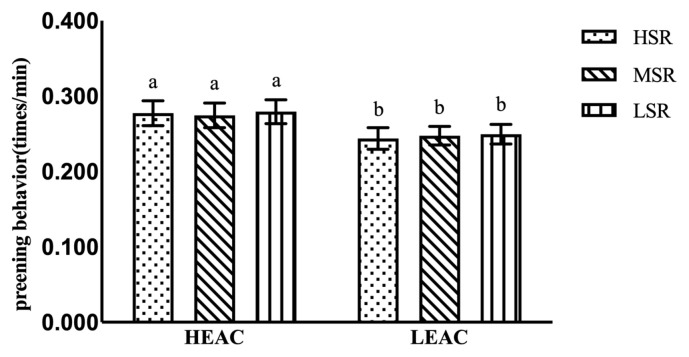
Effects of hen’s social ranking order (SRO) on preening behavior in high and low enrichments allocation cages (HEAC and LEAC). ^a,b^ p<0.01.

As it is shown in Table 6, the EA did not affect general state behaviors of the hens (all p>0.05) except standing (p<0.01) shown as Figure 2 and perching behavior (p<0.01). The EA did not affect general event behaviors of the hens (all p>0.05) except preening behavior (p<0.05), shown as Figure 3, the hens in HEAC showed less standing and more preening behavior than those in LEAC. While SRO had no significant effect on all general behaviors of the hens except perching behavior.

## DISCUSSION

### The impact of social ranking order, perch and dust-bath allocation on perching behavior

Hens perform perching behavior with the allocation of perch in furnished cages [22]. Favati et al [23] pointed out that individuals with dominant social status would be proactive in expressing their perching behavior rather than the passive expression of perching behavior. When the social status of an individual changed, it did not affect the motivation or tendency of hens, but only changed behavior expression [24]. In this experiment, all individuals with different SRO hens performed perching behavior, and that the perching behavior of the HSR was significantly higher than the LSR, which was coincident with partial of our hypothesis. The early experience of using perch may be another factor determining perching desire of hens [25,26]. Therefore, Gunnarsson et al [27] suggested that the hens should be trained to use perch at the breeding period, so that perch can be used more skillfully and perch utilization would be increased [28]. At the start of the experiment, all hens had no early experience of perching, thus it was impossible for the results of this study to verify whether early experience affected the use of the perch. However, even without early perching experience, the hens performed perching behavior. This showed that perching behavior is instinct, and it was not necessarily related to their early experience of perching, as shown in our results that inexperience did not affect perching behavior of the hens.

Although the dominant hens performed more perching behaviors on perch than the lower social status, all individuals in the cage had chances to perform perching behavior. The results showed that due to the small group of laying hens in this experiment, the competition for perch was relatively low, and all individuals had the opportunity to use the perch. Besides, the perching behavior shown by all individuals suggests that hens had a strong desire to perch, and it seemed related to rest behavior pattern for poultry [15]. Cordiner and Savory [29] reported that the LSR might also use their perch as a tool to evade attacks of other hens, resulting in differences in perch-occupancy among the individuals of different social status. LSR were also reported to often use perch to avoid HSR [27]. This conclusion could not be verified because this study did not research on this problem.

### The impact of social ranking order, perch and dust-bath allocation on dustbathing behavior

Shimmura et al [16] found that in their experiments the HSR in large-scale furnished cages showed more frequent dust bathing and bill raking behavior than the LSR, but there was no difference in small-scale furnished cage. In large cage conditions, the HSR would give priority to dust bathing when there was less resource per hen, whereas the LSR would exhibit extremely low nesting but spent more time in using the nest box as a refuge [29,30]. Shimmura et al [5] also reported that the enrichments utilization by the hens in furnished cage could be influenced by their SRO, which also existed in medium - scale furnished cage (10/cage) when the cage enrichments were abundant for all individuals. In large furnished cages, the hens with HSR still showed their right to prioritize the utilization of enrichments, even though these enrichments were sufficient [3]. This suggests that it was determined by the social nature of laying hens rather than enrichments abundance. In this experiment, the hens showed less dustbathing than their perching, and the number of hens using dust-baths was also few, and the individual’s SRO had no effect on dustbathing behavior, but the amount of dust-baths allocation had significant effect on dustbathing behavior. This agreed with results of Shimmura et al [19] in the small-scale furnished cage but was inconsistent with the findings of its large-scale furnished cage. It can be inferred that the effect of social nature of hens on enrichments utilization in the small-scale furnished cages is not as significant as that in the large-scale cages, it may be related to the intensity of social competition for enrichments. Although there is social competition in small furnished cage, the competition may be lower compared with the large furnished cage, the enrichments utilization results will be different. In this study, the experimental cage belonged to the small furnished cage, the flock of laying hens was small, and the dust-baths in the cage were relatively adequate, but dustbathing behavior of hens was rather less, which could not be fully explained in this study, and needs further exploration. However, it can be preliminarily speculated that this may be related to the early experience of perching, for all experimental hens had no dustbathing experience prior to the experiment, and the dustbathing behavior had a social facilitation effect on each other [31].

It has been reported that the area of dust-baths may affect dustbathing behavior of hens, as the hens constantly changed their posture during dustbathing process, and narrow space was not conducive to the hens to perform dustbathing behavior [32]. Shimmura et al [19] pointed out that laying hens of different SRO had significant differences in dustbathing [29]. This was in contrast with our results where the hens with different SRO did not show significant difference in dustbathing behavior but showed a significant difference in perching behavior. The inconsistent result between Shimmura’s and our experiments was possibly due to different experimental conditions, where in Shimmura’s [19] experiment the dust-baths were located above the nest box, contrarily, in our experiment the dust-baths were located at the bottom of the cage.

### The impact of social ranking order, perch, and dust-bath allocation on general behavior

The stimulation of social environment may affect the behaviors of laying hens in cages [33]. When furnished facilities are provided in the cage, the behaviors of laying hens will be affected. Moreover, some studies have shown that the performance of laying hens in enriched cages will be more diversified [19,34]. Feeding and drinking behavior are the basic behavior of animals to maintain growth, oviposition and other needs. Some studies have shown that when there is not enough feed trough allocation for each hen, especially for the LSR, they will be prevented from feeding by the dominant hens in the same cage, so they will be negatively affected [35]. There was no significant effect on feeding and drinking behaviors of layers of different SRO, which indicated that the allocation of feed troughs and water nipples provided in the present experiment met the requirements of EU, which were sufficient for six layers.

The results showed that HEA and LEA did not affect general behaviors but had a significant effect on the performance of standing and preening behaviors of laying hens. This can be easily understood because the same cage space with EA made hens have less space except perch and dust-bath which allowed them to be less active. Different EA may be the reason why there was a significant difference in standing behavior. Meanwhile all laying hens have established a stable social hierarchy and less aggressive hens which maybe have a strong motivation to preen. Eskeland et al [36] found that there was a negative correlation between the dominance of cage birds and the preening, which may be since the less aggressive hens were more likely to be motivated to preen, and they were more used to gathering. While there was no significant difference in general behavior among the layers of different SRO in this study, it may be due to the cage space being large enough for the small group of 6 hens. Different SRO and enrichments had no significant effect on the head shaking behavior of laying hens in this study, which was similar to Nicol’s [37] results that there was no direct correlation between head shaking and dominance.

## CONCLUSION

The results suggest that SRO has a significant effect on the perching behaviors with either HEAC and LEAC, and the hens with the HSR have a priority to use perches; SRO and EA have little effect on dust-bathing and general behaviors.

## Figures and Tables

**Table 1:** Behavioral categories and definitions

Behavioral categories	Definitions
Aggressive pecking	Pecking at head, back or neck except severe pecks (forceful pecks, sometimes with feathers being pulled out and with the recipient bird moving away) and gentle pecks (careful pecks, not resulting in feathers being pulled out and usually without a reaction from the recipient bird)
Threatening	The hen tries to peck the pecked hen, but the pecked hen leaves before being pecked
Displacing	The hen pecks the other hen’s feathers on its back with beak, or steps on back with claws, and finally takes up the position of the other hen
Chasing	The hen chases the other hen until the other hen runs into the corner and could not keep chasing

Behavioral definitions from Appleby [18]; Shimmura et al [19].

**Table 2:** Definitions of perching behavior

Behavioral categories	Definitions
State behaviors	
Standing	Both legs are straightened on the perch
Lying	Lying once posture is lost and not perceived to be purposefully controlling posture
Walking	A hen raises one of its legs with the other leg standing on floor and moves forward
Event behaviors	
Preening	Hen directs its beak to its own plumage of several body parts (thorax, abdomen, shoulder, interior and exterior wings, rump, back, and cloaca) and carries out pecking, nibbling, combing or rotating movements, once or repeatedly
Staring	Hen’s head stays immovability with its eyes open when on a perch
Exploring	Hen’s beak contacts with the perch
Comforting	Behaviors including scratching, body shaking, tail shaking, wing flapping, wing-leg-stretching and wing lifting when hen is on a perch

Brender et al [38]; Casey-Trott and Widowski [39].

**Table 3:** Definitions of dustbathing behavior

Behavioral categories	Definitions
State behaviors	
Head forward	The head of the hen is visible. The neck is angled down- or upwards. The hen is sitting or standing and does not show any body movement
Head under wing	The head of the hen is tucked backwards under the wing or attached laterally to the body. The beak and the crown touch the plumage. The hen is sitting or standing and does not show any body movement
Event behaviors	
Bill raking	Flank friction with beak on the litter
Pecking	Hen’s beak contacts with the litter repeatedly
Vertical wing shaking	Lying in the litter area, the wings beat up and down rhythmically
Side scratching	With the vertical shaking, the head and legs stretch to one side continuously, and dig the litter
Vigorous body shaking	Stand up in the bedding and shake the body or wings
Body movement	The hen shows body movement like head shaking, standing up, sitting down, comfort behavior or balance movements

Brender, et al [38]; Casey-Trott and Widowski [39].

**Table 4:** Definitions of general behavior

Behavioral categories	Definitions [38,39]
State behaviors	
Feeding	The chick is next to the feeder with its head above the food
Standing	Standing on both feet without showing another defined behavior
Lying	Lying once posture is lost and not perceived to be purposefully controlling posture
Walking	Walking more than 2 steps
Perching	Laying hen with two feet on a perch for more than 3s, including standing, sitting, and walking
Dustbathing	Including head forward and head under wing in the dust-bath
Event behaviors	
Drinking	The chick’s bill is oriented to the cup drinkers, while not further away than 5 cm
Preening	Standing or sitting turning the head and start manipulating feathers of the body using the beak
Staring	Hen’s head stays immovability with its eyes open when it is on a perch
Pecking	Moving head backwards and forwards in a pecking motion
Comforting	Behaviors including scratching, body shaking, tail shaking, wing flapping, wing-leg-stretching and wing lifting when hen on a perch
Head shaking	Rapid lateral head movement
Feather pecking	Pecking at feathers of another hen
Escaping	Running away in case of attack or threat

Brender et al [38]; Casey-Trott and Widowski [39].

**Table 5:** Effects of social ranking order on perching behavior in high or low perch and dust-bath allocation (mean±standard deviation)

Grouping	HSR		MSR		LSR		p-value (mean)		
			
	HEAC	LEAC	HEAC	LEAC	HEAC	LEAC	SRO	Enrichment	Interaction
Standing (%)	0.39^a^±0.15	0.30^a^±0.18	0.24^b^±0.11	0.24^b^±0.16	0.22^b^±0.14	0.20^b^±0.16	<0.01	0.06	0.16
Lying (%)	0.21^x^±0.12	0.18^y^±0.16	0.23^x^±0.13	0.11^y^±0.10	0.17^x^±0.18	0.14^y^±0.17	0.19	<0.01	0.09
Walking (%)	0.04^a^±0.03	0.04^a^±0.02	0.02^b^±0.01	0.01^b^±0.01	0.00^c^±0.00	0.00^c^±0.00	<0.01	0.38	0.64
Preening (times/min)	0.45^a^±0.18	0.43^a^±0.26	0.33^b^±0.12	0.36^b^±0.25	0.23^c^±0.20	0.30^c^±0.26	<0.01	0.34	0.32
Staring (times/min)	0.03^a^±0.02	0.03^a^±0.02	0.02^b^±0.01	0.02^b^±0.01	0.01^c^±0.01	0.02^c^±0.01	<0.01	0.19	0.07
Exploring (times/min)	0.07^a^±0.04	0.06^a^±0.05	0.04^b^±0.02	0.04^b^±0.03	0.02^c^±0.02	0.03^c^±0.02	<0.01	0.25	0.11
Comforting(times/min)	0.06^a^±0.04	0.04^a^±0.04	0.03^b^±0.02	0.04^b^±0.04	0.02^c^±0.01	0.02^c^±0.03	<0.01	0.11	0.14

HSR, high social ranking hens; MSR, middle social ranking hens; LSR, low social ranking hens; HEAC, high enrichments allocation cages; LEAC, low enrichments allocation cages; SRO, social ranking order.

a–c Means with different superscripts in a row indicate significant differences on different social ranking order (p<0.01).

x,y Means with different superscripts in a rank indicate significant differences between enrichment treatments (p<0.01).

**Table 6:** Effects of social ranking order on general state behaviors in high or low perch and dust-bath allocation (mean±standard deviation)

Grouping	HSR		MSR		LSR		p-value (mean)		
			
	HEAC	LEAC	HEAC	LEAC	HEAC	LEAC	SRO	Enrichment	Interaction
General behaviors									
Feeding (%)	0.17±0.09	0.21±0.12	0.21±0.12	0.22±0.09	0.22±0.12	0.18±0.09	0.64	0.88	0.29
Standing (%)	0.29^y^±0.14	0.42^x^±0.19	0.29^y^±0.15	0.41^x^±0.17	0.24^y^±0.13	0.38^x^±0.21	0.19	<0.01	0.92
Lying (%)	0.03±0.04	0.04±0.10	0.02±0.03	0.03±0.05	0.03±0.04	0.02±0.04	0.35	0.39	0.38
Walking (%)	0.01±0.01	0.01±0.01	0.01±0.01	0.01±0.01	0.01±0.00	0.01±0.01	0.40	0.50	0.42
Perching (%)	0.64^a,x^±0.22	0.51^a,y^±0.27	0.48^b,x^±0.20	0.36^b,y^±0.21	0.39^b,x^±0.24	0.34^b,y^±0.23	<0.01	0.01	0.44
Dustbathing (%)	0.01±0.03	0.01±0.02	0.01±0.03	0.00±0.01	0.01±0.03	0.01±0.01	0.92	0.08	0.96

HSR, high social ranking hens; MSR, middle social ranking hens; LSR, low social ranking hens; HEAC, high enrichments allocation cages; LEAC, low enrichments allocation cages; SRO, social ranking order.

a–c Means with different superscripts in a row indicate significant differences on different social ranking order (p<0.01).

x,y Means with different superscripts in a rank indicate significant differences between enrichment treatments (p<0.01).
